# Complete Genome Sequence of KPC-3- and CTX-M-15-Producing *Klebsiella pneumoniae* Sequence Type 307

**DOI:** 10.1128/genomeA.00213-16

**Published:** 2016-04-07

**Authors:** Laura Villa, Claudia Feudi, Daniela Fortini, Michele Iacono, Celestino Bonura, Andrea Endimiani, Caterina Mammina, Alessandra Carattoli

**Affiliations:** a Istituto Superiore di Sanità, Rome, Italy; b Roche Tissue Diagnostic & Sequencing, Roche Diagnostic SPA, Monza, Italy; c University of Palermo, Palermo, Italy; d Institute of Infectious Diseases, University of Bern, Bern, Switzerland

## Abstract

*Klebsiella pneumoniae* sequence type (ST) 307, carrying *bla*_KPC-3_, *bla*_CTX-M-15_, *bla*_OXA-1_, *aac(6′)-Ib-cr*, and *qnrB*1 genes, is replacing the predominant hyperepidemic ST258 clone in Italy. Whole-genome and complete plasmid sequencing of one ST307 strain was performed and new features were identified.

## GENOME ANNOUNCEMENT

KPC carbapenemase-producing *Klebsiella pneumoniae* of sequence type (ST) 258 and related variants (Clonal Group CG258) emerged during the early 2000s in the United States and have spread throughout the world (1). A countrywide survey on carbapenem nonsusceptible *K. pneumoniae* isolates showed that the most frequent clones circulating in Italian hospitals belonged to CG258 (2). Specific IncF plasmids with *bla*_KPC_ (pKpQIL) and virulence traits (pKPN3) have contributed significantly to the success of CG258 (3, 4). In more recent surveillance studies from South Italy, multifocal dissemination of KPC-3-producing *K. pneumoniae* (KPC-3-Kp) clones was observed, showing the rapid emergence of the KPC-3-Kp ST307 clone, also coproducing the CTX-M-15 extended spectrum beta-lactamase (ESBL). Here, we report the whole-genome sequencing (WGS) of the Kp-48 ST307 strain, identified from urine-culture in a tertiary care hospital in Palermo, Italy, in May 2014 (5).

WGS was performed on the 454-GS Junior platform according to the standard sequencing procedure (Roche Diagnostics). Genomic coverage was 70×. Reads were aligned and assembled using gsAssembler v2.8 (Roche Diagnostics).

The complete sequence of Kp-48 revealed that the assembled genome size was 5,687,551 bp in length. Sequencing identified 4,395 annotated genes, 76 tRNAs, 25 rRNAs, and 719 open reading frames encoding uncharacterized proteins. Three extrachromosomal elements were found: the two IncFIIK plasmids pKpQIL (116,499 bp) and pKN3 (222,588 bp), and a novel phage (51,998 bp). The *bla*_KPC-3_ gene was localized on the pKpQIL plasmid, 99% identical to that described in ST258 isolates (4). The ST307 strain carries a novel variant of the pKN3 plasmid with the *bla*_CTX-M-15_, *bla*_OXA-1_, *aac(6′)-Ib-cr*, and *qnrB1* resistance genes and two novel virulence clusters, encoding the enzymes for glycogen synthesis and a new urea transport system. An HK97-like phage was also present in the ST307 chromosome. The wzi-173 capsular antigen allele for this strain was exacerbated from the genome sequence, assigning ST307 to the previously described KN2 capsular type (6). Interestingly, the ST307 genome also carried a second capsular cluster, whose best match was found with the *Enterobacter aerogenes* genomes (GenBank accession numbers CP011574, FO203355, and CP002824). Putative VirB type IV secretion were identified in regions of major discordance with the ST258 genome (7).

The ST307 clone, carrying a novel assortment of resistance genes and new plasmid located virulence traits, may compete and prevail with the worldwide successful ST258 clone, further spreading the life-threatening KPC-producing *K. pneumoniae* isolates.

### Nucleotide sequence accession number.

The whole-genome shotgun project of the KP-48 strain has been deposited at DDBJ/EMBL/GenBank under the accession no. LKAB00000000.
